# High B7-H3 expression with low PD-L1 expression identifies armored-cold tumors in triple-negative breast cancer

**DOI:** 10.1038/s41523-024-00618-6

**Published:** 2024-01-27

**Authors:** Jie Mei, Yun Cai, Hongjun Zhu, Ying Jiang, Ziyi Fu, Junying Xu, Lingyan Chen, Kai Yang, Jinlu Zhao, Chenghu Song, Yan Zhang, Wenjun Mao, Yongmei Yin

**Affiliations:** 1 Department of Oncology, The First Affiliated Hospital of Nanjing Medical University, Nanjing, 211166 China; 2 The First Clinical Medicine College, Nanjing Medical University, Wuxi, 214023 China; 3 Department of Oncology, The Affiliated Wuxi People’s Hospital of Nanjing Medical University, Wuxi, 214023 China; 4 Wuxi Maternal and Child Health Care Hospital, Wuxi Medical Center of Nanjing Medical University, Wuxi, 214023 China; 5 Department of Oncology, Nantong Third People’s Hospital Affiliated to Nantong University, Nantong, 226006 China; 6 Department of Gynecology, The Obstetrics and Gynecology Hospital Affiliated to Jiangnan University, Wuxi, 214023 China; 7 Department of General Surgery, The Fourth Affiliated Hospital of Harbin Medical University, Harbin, 150001 China; 8 Department of Thoracic Surgery, The Affiliated Wuxi People’s Hospital of Nanjing Medical University, Wuxi, 214023 China; 9 Jiangsu Key Lab of Cancer Biomarkers, Prevention and Treatment, Collaborative Innovation Center for Personalized Cancer Medicine, Nanjing Medical University, Nanjing, 211166 China

**Keywords:** Breast cancer, Diagnostic markers

## Abstract

Triple-negative breast cancer (TNBC) is generally regarded as the most aggressive subtype among breast cancers, but exhibits higher chemotherapeutic and immunotherapeutic responses due to its unique immunogenicity. Thus, appropriate discrimination of subtypes is critical for guiding therapeutic options in clinical practice. In this research, using multiple in-house and public cohorts, we investigated the expression features and immuno-correlations of B7-H3 in breast cancer and checked the anti-tumor effect of the B7-H3 monoclonal antibody in a mouse model. We also developed a novel classifier combining B7-H3 and PD-L1 expression in TNBC. B7-H3 was revealed to be related to immuno-cold features and accumulated collagen in TNBC. In addition, targeting B7-H3 using the monoclonal antibody significantly suppressed mouse TNBC growth, reversed the armored-cold phenotype, and also boosted anti-PD-1 immunotherapy. In addition, patients with B7-H3 high and PD-L1 low expression showed the lowest anti-tumor immune infiltration, the highest collagen level, and the lowest therapeutic responses to multiple therapies, which mostly belong to armored-cold tumors. Overall, this research provides a novel subtyping strategy based on the combination of B7-H3/PD-L1 expression, which leads to a novel approach for the management of TNBC.

## Introduction

Based on the latest statistical data, breast cancer (BC) is the most prevalent cancer type in the world nowadays, surpassing lung cancer^1^. Being a heterogeneous disease, BC can be classified into four molecular subtypes depending on the status of the hormone receptor (HR) and the human epidermal growth factor receptor 2 (HER2)^2^. Among all subtypes, triple-negative breast cancer (TNBC) is generally regarded as the most aggressive subgroup with rapid progression, limited therapeutic targets, and unfavorable clinical outcomes^3,4^. Immune checkpoint inhibitors (ICIs) have changed the therapeutic landscape for TNBC over the past 10 years, due to the unique immunogenicity of this BC subtype^5,6^. However, not all patients with TNBC could benefit from anti-PD-1/PD-L1 therapy. Thus, the identification of appropriate biomarkers assessing the characteristics of the tumor immune microenvironment (TIME) is essential for therapeutic options.

A developing immune checkpoint molecule known as B7 homolog 3 protein (B7-H3), also known as CD276, plays a dual role in the immune system^7^. The B7-H3 protein is very infrequently seen in the majority of normal human tissues, but it is consistently overexpressed in a majority of tumor tissues^8^. High B7-H3 expression has been found in BC, and these levels are substantially linked with greater tumor size and lymphatic invasion^9,10^. In addition to BC, B7-H3 has been proven to be highly expressed in papillary thyroid carcinoma, glioma, non-small cell lung cancer, melanoma, and head and neck squamous cell carcinoma^11–13^. Although the role of B7-H3 is dual, B7-H3 is well-known to negatively modulate cytotoxic T lymphocytes (CTLs)-mediated cancer immunity and inhibition of B7-H3 could increase the activity of CTLs^14^. These findings indicate that anti-B7-H3 therapy is appropriate for cancer patients with B7-H3 overexpression. Multiple anti-B7-H3 therapies have been proposed, such as DS-7300, MGA271, MGD009, and B7-H3 chimeric antigen receptor T-cell immunotherapy (CAR-T)^15^. However, the expression of B7-H3 in TNBC and its value as a biomarker to identify therapeutic options have not been elucidated.

In this study, using multiple in-house and public cohorts, we investigated B7-H3’s expression characteristics in BC and discovered associations between B7-H3 and immuno-cold characteristics and accumulated collagen in TNBC. TNBC patients with B7-H3 high and PD-L1 low expression showed the lowest therapeutic responses to multiple therapies. Overall, we provide a novel subtyping strategy based on the combination of B7-H3/PD-L1 expression, thereby contributing to the improvement of treatment for TNBC.

## Results

### Characterization of B7-H3 expression in BC

**Fig. 1:**
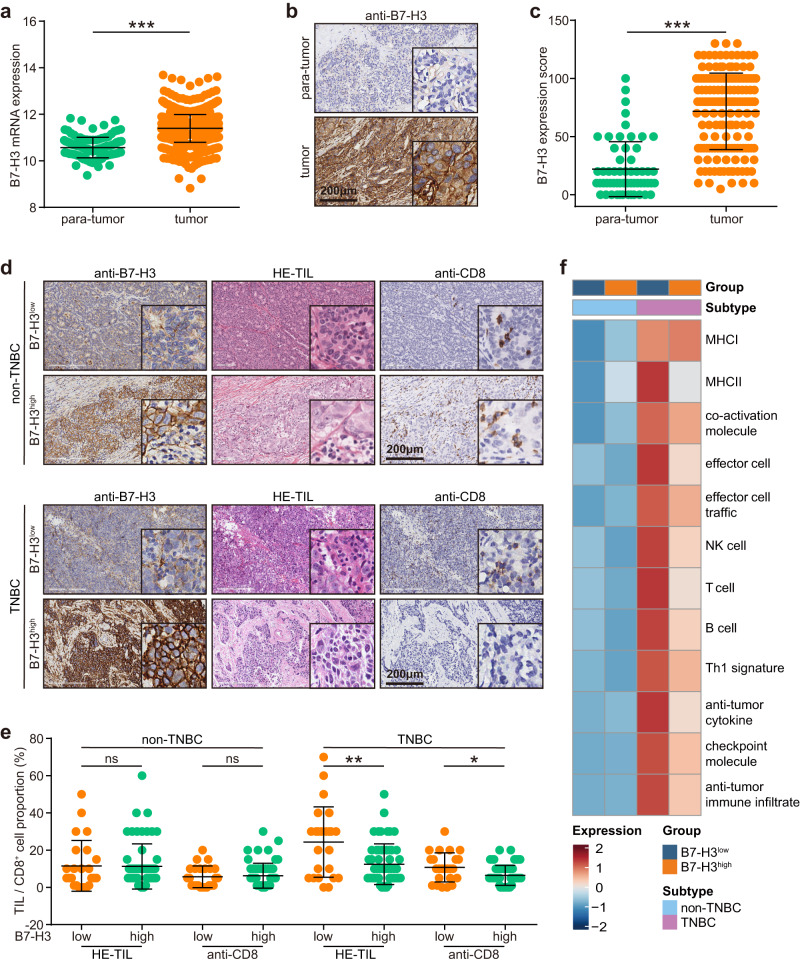
Expression pattern of B7-H3 in BC. **a** Transcriptional expression of B7-H3 para-tumor (*n* = 114) and tumor (*n* = 1104) tissues in the TCGA cohort. Data presented as mean ± SD. Significance was calculated with Student’s *t* test. **b**, **c** Representative images uncovering B7-H3 expression in para-tumor (*n* = 6) and tumor (*n* = 6) tissues and quantitative analysis. Significance was calculated with paired Student’s *t* test. **d**, **e** Representative images uncovering TILs and CD8^+^ T cells infiltration in non-TNBC and TNBC samples with the low and high B7-H3 expression and quantitative analysis. *n* (samples with low B7-H3 in non-TNBC) = 24, *n* (samples with high B7-H3 in non-TNBC) = 59, *n* (samples with low B7-H3 in TNBC) = 24, and *n* (samples with high B7-H3 in TNBC) = 56. Data presented as mean ± SD. Significance in non-TNBC samples was calculated with Student’s *t* test, and significance in TNBC samples was calculated with Mann–Whitney test. **f** Anti-tumor immune infiltration levels in non-TNBC and TNBC samples with the low and high B7-H3 expression in the TCGA cohort.

We first characterized the expression of B7-H3 in BC. Compared with normal breast tissues, B7-H3 was highly expressed in tumor tissues in the in-house and the Cancer Genome Atlas (TCGA) cohorts (Fig. 1a–c). We also compared B7-H3 expression in BC with different subtypes. Tumors with high B7-H3 expression accounted for 70.08% (59/83) in non-TNBC samples, and 70.00% (56/80) in TNBC samples. B7-H3 expression did not differ significantly between non-TNBC and TNBC samples in the in-house and the TCGA cohorts, except for the METABIRC cohort (Supplementary Fig. 1a–c). The relationships between B7-H3 and immune infiltration were also examined in non-TNBC and TNBC samples. Given that the proportion of B7-H3 high expression was 7.00% in the in-house TNBC cohort, we set 70.00% as the cut-off value for B7-H3 in public cohorts. There was no notable difference in tumor-infiltrating lymphocytes (TILs) and CD8^+^ cells infiltration between B7-H3 low and high expressed non-TNBC samples (Fig. 1d, e), but TILs and CD8^+^ cells was notably higher in the B7-H3 low expressed TNBC samples (Fig. 1d, e). Similar findings were also observed in the TCGA and the METABIRC cohorts (Fig. 1h and Supplementary Fig. 1d). In addition, we also compared B7-H3 expression in primary and metastatic TNBC tissues. The results showed that B7-H3 was unchanged in primary and metastatic tissues. Given the negative correlation between B7-H3 and immune infiltration, we defined immuno-hot metastasis (lung and pleura) and immune-cold (liver, brain, bone, and skin) metastasis according to previous research^16,17^, and we found that B7-H3 was highly expressed in immune-cold metastatic sites compared with primary tissues (Supplementary Fig. 2a, b). Overall, these results suggest B7-H3 does not have difference in expression in non-TNBC and TNBC, but is specifically correlated with decreased immune cells infiltration in TNBC.

### B7-H3 is correlated with collagen index and upregulated in “armored-cold” TNBC

**Fig. 2:**
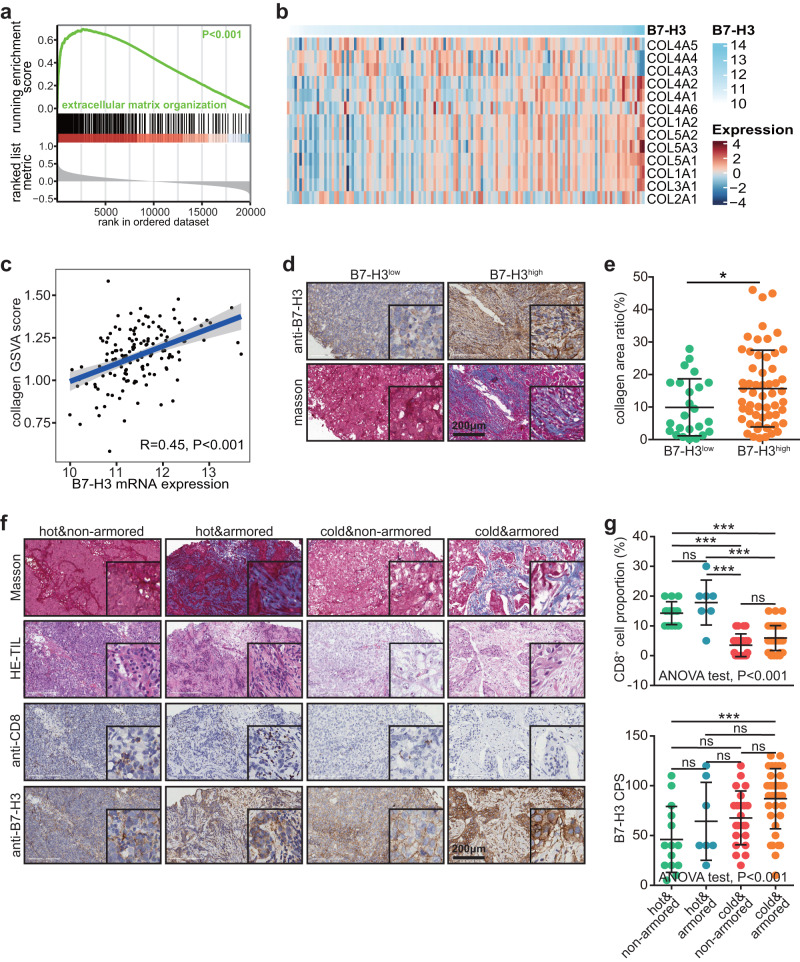
Correlation between B7-H3 and collagen deposition in TNBC. **a** GSEA revealing the association between B7-H3 and extracellular matrix organization. **b**, **c** Correlation between B7-H3 and collagen gene expression and collagen GSVA score. Significance was calculated with Pearson test. **d**, **e** Representative images uncovering collagen infiltration in TNBC samples with the low (*n* = 24) and high (*n* = 56) B7-H3 expression and quantitative analysis. Data presented as mean ± SD. Significance was calculated with Student’s *t* test. **f**, **g** Representative images uncovering CD8^+^ T cells infiltration and B7-H3 expression in TNBC samples with different subtypes, including “hot & non-armored (*n* = 14)”, “hot & armored (*n* = 7)”, “cold & non-armored (*n* = 22)”, and “cold & armored (*n* = 37), and quantitative analysis. Data presented as mean ± SD. Significance was calculated with one-way ANOVA with Tukey’s multiple-comparison test.

Next, the potential biological functions of B7-H3 in TNBC was explored. The GSEA analysis revealed that B7-H3 was mostly associated with extracellular matrix organization in the TCGA cohort (Fig. 2a). Given that collagen proteins are abundantly present in the extracellular matrix and play significant roles in cancer progression^18^, we next assessed correlations between B7-H3 and collagens, and the results showed that B7-H3 was positively related to most collagens (Fig. 2b, c). Similar findings were also observed in the METABIRC cohorts (Supplementary Fig. 3a, b). In addition, the results from the in-house cohort also indicated that tumors with high B7-H3 expression had more collagen deposition (Fig. 2d, e). Further analysis showed that B7-H3 expression was positively correlated with collagen levels but negatively correlated with immune cells infiltration (Supplementary Fig. 4a, b). Subsequently, we divided tumors into four types based on the immune and collagen features. Tumors with high TILs levels simultaneously without collagen deposition were defined as “hot & non-armored” tumors. Other subtypes named “hot & armored”, “cold & non-armored”, and “cold & armored” were defined in turn (Fig. 2f). CD8^+^ T cells were the lowest, but B7-H3 was the highest in the “cold & armored” tumors, which showed the poorest prognosis and the lowest therapeutic responses in principle (Fig. 2g)^19^. Moreover, we extracted response-related genes in the merged durvalumab-based therapy cohort (Supplementary Fig. 5a), and found that genes associated with the poor response were enriched in the “extracellular matrix organization” (Supplementary Fig. 5b), and genes associated with the well response were enriched in the “immune system” (Supplementary Fig. 5c), indicating extracellular matrix organization significantly hindered the immunotherapy efficacy. Taken together, B7-H3 was highly correlated with collagen deposition in TNBC and overexpressed in “cold & armored” subtypes.

### Targeting B7-H3 inhibits TNBC growth and boosts anti-PD-1 therapy

**Fig. 3:**
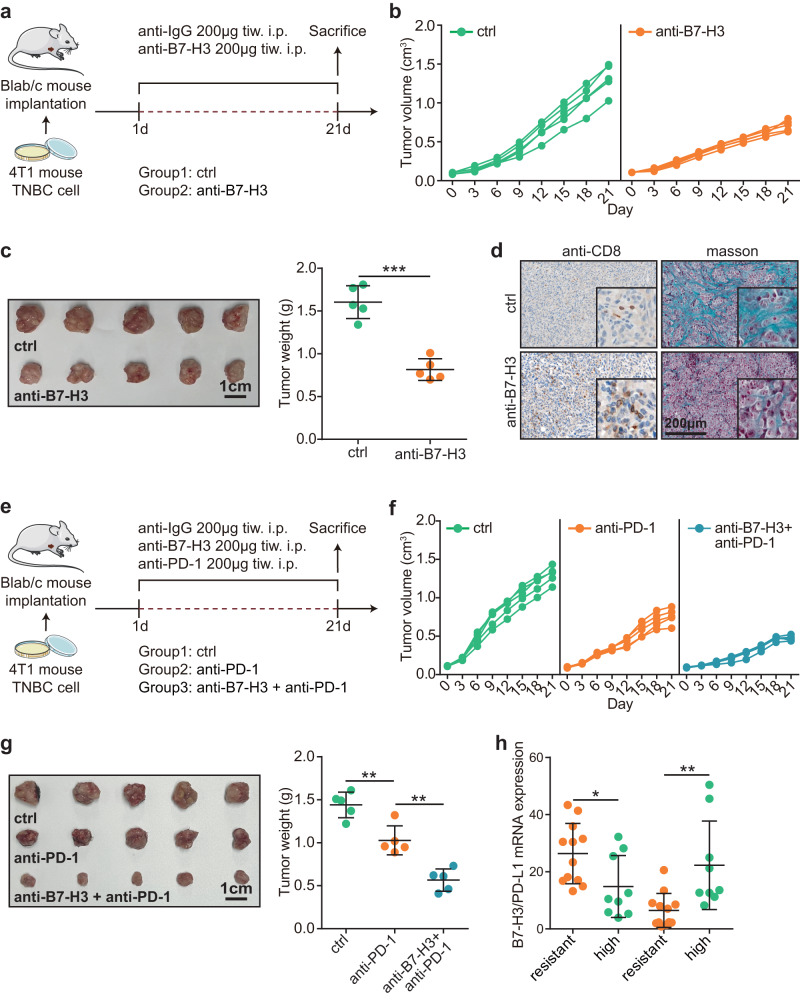
Pharmacological effect of B7-H3 mAb in mouse TNBC model. **a** Schematic protocol of anti-B7-H3 therapy in balb/c mice bearing 4T1 cells. **b** Effect of B7-H3 mAb on tumor volume in balb/c mice bearing 4T1 cells (*n* = 5). **c** Effect of B7-H3 mAb on tumor weight in balb/c mice bearing 4T1 cells (*n* = 5) and quantitative analysis. Data presented as mean ± SD. Significance was calculated with Student’s *t* test. **d** Representative images uncovering the effects of B7-H3 mAb on immune cells and collagen infiltration in balb/c mice bearing 4T1 cells. **e** Schematic protocol of the combination of anti-B7-H3 and anti-PD-1 antibodies in balb/c mice bearing 4T1 cells. **f** Effect of the combination of B7-H3 and PD-1 mAbs on tumor volume in balb/c mice bearing 4T1 cells (*n* = 5). **g** Effect of the combination of B7-H3 and PD-1 mAbs on tumor weight in balb/c mice bearing 4T1 cells (*n* = 5) and quantitative analysis. Data presented as mean ± SD. Significance was calculated with one-way ANOVA with Tukey’s multiple-comparison test. **h** Expression levels of B7-H3 and PD-L1 in immunotherapy-resistant (*n* = 12) and immunotherapy-sensitive (*n* = 9) tumor cell lines. Data presented as mean ± SD. Significance in B7-H3 expression was calculated with Student’s *t* test, and significance in PD-L1 expression was calculated with Mann–Whitney test.

To test the anti-tumor effect of B7-H3 mAb, we used the 4T1 cell line to establish mouse TNBC models (Fig. 3a). Anti-B7-H3 therapy significantly inhibited tumor growth in mouse TNBC model (Fig. 3b, c). Moreover, we found that anti-B7-H3 therapy notably increased immune cells infiltration using anti-CD8 immunohistochemistry (IHC) staining, and also reduced collagen deposition within the tumor (Fig. 3d). Given that B7-H3 mAb could shape immuno-hot tumor immune microenvironment (TIME), we next explored whether B7-H3 mAb could boost anti-PD-1 immunotherapy (Fig. 3e). The results showed that B7-H3 mAb significantly enhanced the anti-tumor effect of anti-PD-1 therapy (Fig. 3f, g). In addition, the results from the GSE168846 dataset suggested that B7-H3 was revealed to be highly expressed in immunotherapy-resistant tumor cell lines and PD-L1 was lowly expressed in immunotherapy-resistant tumor cell lines (Fig. 3h), supporting that B7-H3 was associated with the immuno-suppressive TIME. Overall, B7-H3 is an emerging target to inhibit tumor growth in TNBC, overcome “armored-cold” TIME features, and ultimately boost anti-PD-1 therapy.

### Combination of B7-H3 and PD-L1 divides TNBC samples into three subtypes

**Fig. 4:**
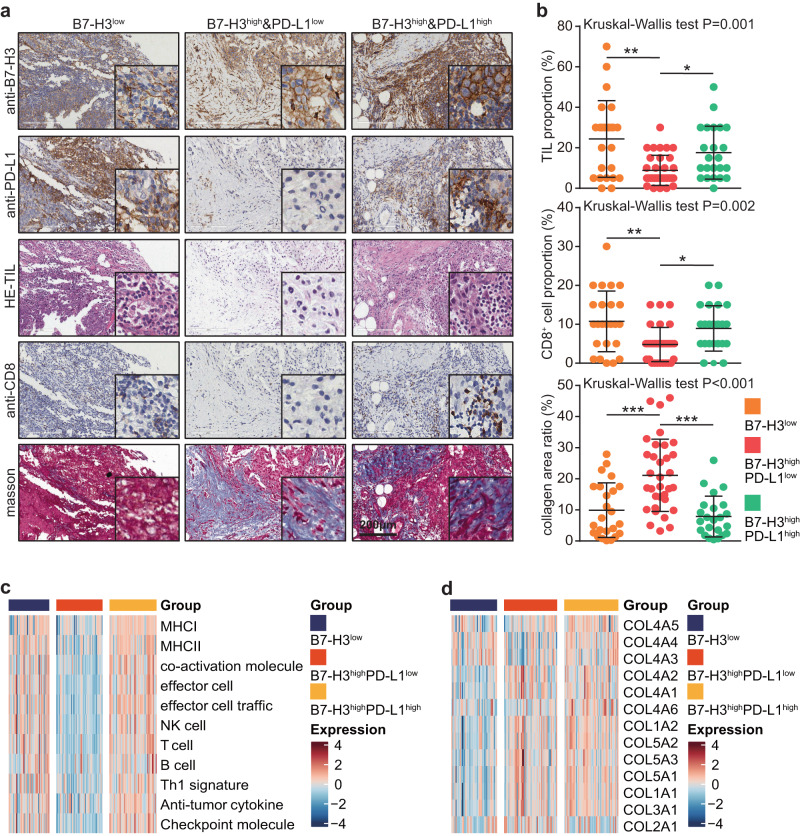
TIME features of three subtypes divided by the combination of B7-H3/PD-L1. **a**, **b** Representative images uncovering immune cells and collagen infiltration in TNBC samples in the B7-H3^low^ (*n* = 24), the B7-H3^high^&PD-L1^low^ (*n* = 33), and the B7-H3^high^&PD-L1^high^ (*n* = 23) subtypes and quantitative analysis. Data presented as mean ± SD. Significance was calculated with Kruskal–Wallis with Dunn’s multiple-comparison test. **c** Difference of anti-tumor immune infiltration in TNBC samples in the B7-H3^low^, the B7-H3^high^&PD-L1^low^, and the B7-H3^high^&PD-L1^high^ subtypes in the TCGA cohort. **d** Difference of collagen infiltration in TNBC samples in the B7-H3^low^, the B7-H3^high^&PD-L1^low^, and the B7-H3^high^&PD-L1^high^ subtypes in the TCGA cohort.

Many studies have revealed that B7 molecule patterns that are mutually exclusive or co-expressed can predict whether time is inflamed or not in various malignancies^20,21^. Given the notable value of PD-L1 in predicting immunotherapy responses, we also combined B7-H3 and PD-L1 expression to establish a novel subtyping strategy. We found that TNBC patients with B7-H3^high^PD-L1^low^ feature exhibited the lowest anti-tumor immune infiltration and the highest collagen deposition level (Fig. 4a, b). Given that the proportion of PD-L1 high expression was 48.75% in the in-house TNBC cohort, we set 50% as cut-off value for PD-L1 in public cohorts. In the TCGA and the METABRIC cohorts, similar to the results concluded from the in-house cohort, TNBC patients with B7-H3^high^PDL1^low^ feature exhibited the lowest anti-tumor immune infiltration and higher collagen deposition level (Fig. 4c, d and Supplementary Fig. 6a, e). Overall, the combination of B7-H3 and PD-L1 expression could divide TNBC samples into three subtypes, and the B7-H3^high^PD-L1^low^ subgroup exhibits the “armored-cold” features.

### The B7-H3/PD-L1 classifier is associated with responses to multiple therapies in TNBC

**Fig. 5:**
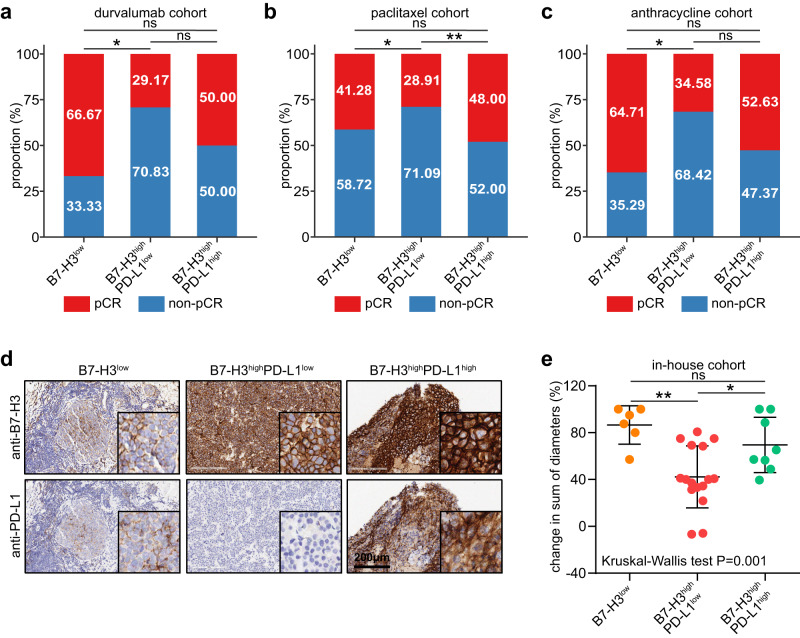
Association between the B7-H3/PD-L1 classifier and therapeutic responses. **a**–**c** Association between the B7-H3/PD-L1 classifier and therapeutic responses in the durvalumab-dependent cohort, the paclitaxel-dependent cohort, and the anthracycline-dependent cohort. Significance was calculated with chi-squared test. **d** Representative images uncovering the discrimination of TNBC samples into the B7-H3^low^ (*n* = 6), the B7-H3^high^&PD-L1^low^ (*n* = 15), and the B7-H3^high^&PD-L1^high^ (*n* = 9) subtypes in the recruited NAT cohort. **e** Association between the B7-H3/PD-L1 classifier and therapeutic responses in the recruited NAT cohort. Data presented as mean ± SD. Significance was calculated with one-way ANOVA with Tukey’s multiple-comparison test.

Given that TIME features were related to response to multiple therapies in TNBC^22^, we examined the association between the B7-H3/PD-L1 classifier and therapeutic responses. In three public cohorts, including the durvalumab-dependent cohort, the paclitaxel-dependent cohort, and the anthracycline-dependent cohort, the B7-H3^high^PDL1^low^ subgroup exhibited the lowest therapeutic response (Fig. 5a–c). In addition, in the above three cohorts, the B7-H3^high^PD-L1^low^ subgroup exhibited the lowest anti-tumor immune infiltration and higher collagen deposition level (Supplementary Fig. 6b–d, f–h), namely the “armored-cold” feature. Moreover, in our recruited NAT cohort, we found that the B7-H3^high^PD-L1^low^ subgroup also had the lowest therapeutic response (Fig. 5d, e). Overall, the B7-H3/PD-L1 classifier is associated with therapeutic responses to multiple therapies in TNBC patients, and patients with B7-H3^high^PD-L1^low^ face limited therapeutic strategies and poor therapeutic responses.

## Discussion

B7-H3 is a member of the B7 family, and in tumor samples, B7-H3 expression was more frequent than PD-L1 expression in a variety of cancer types^23,24^. Despite the fact that the B7-H3 receptor’s identification is uncertain, accumulating evidence has found that B7-H3 promotes tumor cell aggressiveness in most cases^15^. According to a previous research, B7-H3 suppresses the NF-κB, NFAT, and AP-1 signaling pathways to decrease T cell activity, while inhibition of B7-H3 increases T cell activation in murine models^25^. B7-H3 is substantially expressed in cancer stem cells, and by suppressing B7-H3, CD8^+^ T cell infiltration and tumor inhibition in head and neck squamous cell carcinoma are both dramatically boosted^26^. In addition, B7-H3 is also expressed in cancer-associated fibroblasts (CAFs) and exerts an anti-apoptotic role in CAFs, thus enhancing cancer cell invasion in renal cancer^27^. Overall, B7-H3 modifies the immune response by affecting several TIME features in human malignancies,

B7-H3 has a wide range of possible uses in BC, which is encouraging. B7-H3 enhances tumor-associated macrophages’ pro-angiogenic activity in TNBC^28^. In addition, according to a recent study, the amount of CD3^+^ and CD8^+^ T cell infiltration was adversely linked with the expression of B7-H3 mRNA and protein in tumor cells^29^. In our study, we discovered that whereas B7-H3 expression was not different between non-TNBC and TNBC samples, it was substantially elevated in BC. However, B7-H3 was associated with immuno-cold features in TNBC instead of non-TNBC subtype. Moreover, B7-H3 was associated with accumulated collagen deposition. Combining these two features of the TIME, B7-H3 was highly expressed in “armored-cold” tumors characterized by low immune cells infiltration and high collagen deposition, which exhibited the lowest therapeutic response to immunotherapy^19^. We also found that targeting B7-H3 inhibited TNBC growth and enhanced anti-PD-1 immunotherapy in vivo. Totally, B7-H3 is a promising biomarker to identify “armored-cold” tumors and also a target to overcome the immuno-cold phenotype and immuno-suppression in TNBC.

PD-L1 expression has been used as the most reliable predictive biomarker for anti-PD-1/PD-L1 therapy and the immuno-hot TIME in multiple cancer types, including TNBC^30,31^. A growing number of clinical studies have revealed a significant correlation between high PD-L1 expression in TNBC patients and a high response rate to PD-1/PD-L1 blocking^31^. Nevertheless, the effectiveness of ICIs is not predicted by PD-L1 as a consequence of multiple clinical trials^32,33^. Although the potential predictive value of PD-L1 in TNBC remains controversial, the detection of PD-L1 expression is the major approach to predicting the response, especially when combined with other biomarkers^34^. Based on the levels of B7-H3 and PD-L1 expression, we developed a novel classifier to identify distinct immuno-subtypes and therapeutic options in TNBC. Patients with B7-H3 high and PD-L1 low expression showed the lowest anti-tumor immune infiltration, the highest collagen level, and the lowest therapeutic responses to multiple therapies, which mostly belong to “armored-cold” tumors.

In recent years, targeting B7-H3 as an inhibitory immune checkpoint has been successful in reducing tumor development in preclinical models, which has substantially stoked interest in clinical translation. Multiple completed and ongoing clinical trials targeting B7-H3 revealed satisfactory curative effects in various cancer types^15^. However, the biomarkers screening the dominant population benefiting from anti-B7-H3 therapy have not been defined. Several case reports implied that patients with high B7-H3 expression in tumor tissues were more likely to benefit from the anti-B7-H3 treatment^35,36^. We reported that TNBC patients with B7-H3 high and PD-L1 low expression face unsatisfactory therapeutic responses, urgently need novel therapies. Thus, it is clear that the B7-H3/PD-L1 classifier can contribute to the management of TNBC.

It is true that there are still certain limitations in the study. The major limitation of the current study is that no standardized clinical trial is conducted to compare the therapeutic responses to anti-B7-H3 therapy or conventional chemotherapies in TNBC patients with B7-H3 high along with PD-L1 low expression. In addition, B7-H3 is the therapeutic target for anti-B7-H3 therapy, thus TNBC patients with B7-H3 high along with PD-L1 high expressed may also benefit from the anti-B7-H3 therapy. However, as this population exhibits high responses to conventional treatments, we did not explore it in depth.

In conclusion, we proposed a novel classifier combined B7-H3 and PD-L1 expression in TNBC based on the relationship between B7-H3 expression and TIME features. Anti-B7-H3 therapy could be a significant supplemental therapeutic strategy in TNBC and patients with B7-H3 high along with PD-L1 low expression may be the significant dominant population benefiting from anti-B7-H3 therapy. Overall, more attempts at anti-B7-H3 treatment should be made in TNBC to change the outcome of a subset of patients with a suboptimal response to conventional therapies.

## Methods

### Specimens and tissue microarrays

The paraffin-embedded tissue microarrays (TMAs) of non-TNBC (Cat. HBreD090Bc03), TNBC (Cat. HBreD090Bc01), and para-tumor breast tissues (Cat. HBreD077Su01) were provided by Outdo BioTech (Shanghai, China). Outdo BioTech provided detailed clinic-pathological features. The Clinical Research Ethics Committee of Outdo Biotech granted ethical approval for the use of TMAs. Samples that were exfoliated during IHC, Hematoxylin and Eosin (HE), and masson staining were removed. A total of 163 samples (83 non-TNBC and 80 TNBC) were included in this study. The HE staining was reviewed by two pathologists, and confirmed 59 samples were confirmed to be para-tumor samples. In addition, 30 TNBC patients receiving standardized neoadjuvant chemotherapy (NAT) were recruited from four independent medical units, including The First Affiliated Hospital of Nanjing Medical University, The Affiliated Wuxi People’s Hospital of Nanjing Medical University, Wuxi Maternal and Child Health Care Hospital, and Nantong Third People’s Hospital. In addition, the responses were assessed using the RECIST1.1 criterion after receiving 8 cycles of NAT. The paraffin-embedded samples of these patients before receiving NAT were obtained. Supplementary Table 1 lists the specific clinic-pathological characteristics. Ethical approvals for the collection of the NAT cohort were granted by the Clinical Research Ethics Committee in above involved institutions and this study was conducted in accordance with the Helsinki Declaration. Written informed consent was waived because of the retrospective nature of this study.

### Single-cell RNA-seq data acquisition and analysis

The single-cell RNA-sequencing (scRNA-seq) datasets of triple-negative breast cancer (TNBC) patients were obtained from the GEO database (GSE180286)^37^. Quality control and pre-processing procedures was performed using the “Seurat” (4.0.5, https://satijalab.org/seurat/) R toolkit^38^. To avoid the influence of abnormal cells and technical noise on downstream analysis, we removed the low-quality cells, including doublets and empty droplets. Cells were removed if the expression of mitochondrial genes was greater than 20% or detected genes were less than 200 or greater than 6000. Finally, a total of 6685 cells from two TNBC patients were reserved for further analysis. In order to minimize the technical batch effects among individuals and experiments, we used the “RunHarmony” function in the R package “harmony”^39^ to perform integration. The top 2000 variable genes were used for principal component analysis (PCA) to reduce dimensionality. The dimensionality of the scaled integrated data matrix was further reduced to two-dimensional space based on the first 20 principal components (PCs) and visualized by t-Distributed Stochastic Neighbor Embedding (t-SNE). The cell clusters were identified based on a shared nearest neighbor (SNN) modularity optimization-based clustering algorithm with a resolution of 0.8. According to the expression levels of some well-known markers, the 6685 cells were annotated as six cell types, including epithelial cells (*n* = 48), fibroblasts (*n* = 187), B cells (*n* = 1077), T cells (*n* = 2923), macrophages (*n* = 1213), and tumor cells (*n* = 1237). The expression of B7-H3 in different cell types was then examined.

### Immunohistochemistry, HE, and masson staining

For the tissue slides and TMAs mentioned above, IHC and HE staining were performed according to the standard operating procedures. Anti-B7-H3 (1:20000 dilution, Cat. ab219648, Abcam, Cambridge, UK), anti-PD-L1 (Ready-to-use, 1:1 dilution, Cat. GT2280, GeneTech, Shanghai, China), and anti-CD8 (Ready-to-use, 1:1 dilution, Cat. PA067, Abcarta, Suzhou, China) were the primary antibodies used in the study. With the use of DAB and a hematoxylin counterstain, antibody staining was visualized. TMAs and tissue slides were Masson stained using the trichrome stain (Masson) kit (Cat. FH115100, FreeThinking, Nanjing, China) in accordance with the manufacturer’s instructions to assess the collagen deposition.

### Quantitative evaluation

Two independent pathologists independently assessed the majority of stained sections. After initially reviewing the TMA with the B7-H3 staining, we found that B7-H3 expression was not limited to tumor cells, but also in endothelial and stromal cells, Thus, we used scRNA-seq data (GSE180286)^37^ to analyze the expression patterns of B7-H3 in TNBC, and the results showed that B7-H3-positive cells were mainly composed of tumor cells, fibroblasts, macrophages, and endothelial cells (Supplementary Fig. 7). Higher expression of B7-H3 on endothelial cells was associated with higher grade malignancies and poor survival^40,41^. B7-H3 expression on a subset of cancer-associated fibroblasts in breast cancer has been reported to contribute to T cell skewing toward regulatory functions^42^. In addition, B7-H3 was also expressed in macrophages and related to T cell functions^43^. After the awareness of the broad expression pattern and its critical functions, we used a Combined Positive Score (CPS)-like scoring method to assess B7-H3 expression. Namely, B7-H3 CPS = (the number of membrane-positive tumor cells, immune cells, fibroblasts, and endothelial cells) / (total number of tumor cells) × 100. TNBC samples with B7-H3 CPS ≥ 50 were deemed to be highly expressed. For quantitative evaluation of the PD-L1 staining, the CPS scoring method was used. TNBC samples with PD-L1 CPS ≥ 10 were deemed to be highly expressed. The fraction of TILs was assessed using the HE staining and CD8^+^ T cells were assessed using the anti-CD8 staining according to previous research^44^. For masson staining, the percentages of positive-stained area were calculated by a senior pathologist with the assistance of the HALO software. To qualitatively distinguish the level of TILs, a hierarchical TIL score was also estimated^45^. Samples with the TIL score ≥3 were considered immune-infiltrated^45^, and samples with the percentage of positive-stained collagen area ≥10% were considered collagen-rich.

### Animal models

Five to six week old female BALB/C mice were obtained from Shanghai SLAC Laboratory Animal Co. Ltd. The mice were given unrestricted access to food and water while being maintained in specialized pathogen-free (SPF) grade experimental animal facilities. The mice were kept in a 12-h light/dark cycle at a temperature of 22 °C ± 2 °C and 70% relative humidity. All experimental procedures were complied with all relevant ethical regulations for animal testing and research. All of the mouse studies were approved by the Laboratory Animal Ethics Committee of Nanjing Medical University. To establish the mouse TNBC model, 4T1 (Cat. KG338, KeyGENE, 5 × 10^6^) cells were subcutaneously injected into the flanks of BALB/C mice. Every 2–3 days, calipers were used to measure and monitor the tumors. When tumors reached about 100 mm^3^ in volume, BALB/C mice bearing 4T1 cells were randomized into two groups (*n* = 5), including control and anti-B7-H3 groups. The B7-H3 in vivo monoclonal antibody (mAb) (Cat. BE0124, BioXCell) and the IgG1 isotype control (Cat. BE0088, BioXCell) was dissolved in InVivoPure™ pH 7.0 dilution buffer, and 200 μg was administered intraperitoneally 3 times a week. The course of therapy was maintained for another 21 days or until the tumors measured 20 mm along their long axis. Mice were euthanized using carbon dioxide (Euthanex Chamber). The tumors were removed from the unconscious animals, which was subsequently documented and weighed. The removed tumors were sent for IHC and masson staining. CD8 antibody (1:1000 dilution, Cat. ab217344, Abcam) was incubated on the sections at 4 °C for an overnight period before secondary antibodies were added.

In addition, we also investigated the therapeutic effectiveness of the combination of anti-B7-H3 and anti-PD-1 therapy. Similar to the method described above, when tumors reached about 100 mm^3^ in volume, BALB/C mice bearing 4T1 cells were randomized into 3 groups (*n* = 5), including control, anti-PD-1, and anti-B7-H3 + anti-PD-1 groups. PD-1 mAb (Cat. BE0273, BioXCell) was dissolved in InVivoPure™ pH 7.0 dilution buffer, and 200 μg was administered intraperitoneally 3 times a week.

### Acquisition of public cohorts

Multiple BC and TNBC public cohorts were collected. Through the UCSC Xena data portal (https://xenabrowser.net/datapages/), the standardized RNA-sequencing (RNA-seq) dataset for BC and clinical data were retrieved. Also, the METABRIC cohort’s clinical data and normalized RNA-seq data were obtained from the cBioPortal data portal (http://www.cbioportal.org/datasets)^46^. The public cohorts including therapeutic information were also obtained, including the GSE176307 dataset (durvalumab-based therapy)^47^, the GSE194040 dataset (paclitaxel-based therapy)^48^, the PRJNA558949 dataset (durvalumab-based therapy)^49^, and the GSE34138 dataset (anthracycline-based therapy)^50^. In addition, three datasets including the transcriptomic data from primary and metastatic tissues, including the GSE209998 dataset^51^, the GSE193103 dataset^51^, and the GSE147322 dataset^52^, were downloaded from the GEO database. The primary and paired at least one metastasis were both TNBC were used for analysis. For the incorporation of transcriptomic data from different datasets, we first merged the datasets using the R package “sva”^53^. Furthermore, we moved the batch effects according to reported methods^54^. Moreover, the GSE168846 dataset, including 11 transcriptomic data from syngeneic mouse tumors, was also downloaded from the GEO database^55^. The summary of public and in-house cohorts was showed in Supplementary Table 2.

### Screening of co-expressed genes with B7-H3 and enrichment analysis

The Pearson’s correlations between B7-H3 expression and transcriptional levels of other genes were measured by the R function “cor.test”, and used to generate the ranked list files. Then, the Gene Set Enrichment Analysis (GSEA) was performed using the R package “clusterProfiler” and the Molecular Signature Database (version 2022)^56^.

### Screening of response-related genes and enrichment analysis

The R package “limma” was used for response-related genes screening in the merged durvalumab-based therapy cohort and genes with *p* value < 0.05 were submitted for gene set enrichment analysis. For gene set enrichment analysis, the DAVID online tool was used to conclude the involved pathways of response-related genes in terms of “REACTOME”. Top 5 terms were exhibited in the results.

### Analysis of tumor immune microenvironment features

The characteristics of the TIME were primarily reflected according to the levels of anti-tumor immune infiltration^19^ and the levels of collagen deposition in matrix. Total score of these terms was calculated using the GSVA algorithm^57^. The associations between subtypes based on the combination of B7-H3/PD-L1 and anti-tumor immune infiltrate and collagen deposition were evaluated.

### Statistical analyses

R version 4.0.2 and Graphpad Prism 6.0 were used to conduct all statistical analyses. The statistical difference of continuous variables between the two groups was evaluated by the Student’s *t* test or Mann–Whitney test according to the applicable conditions. The difference between multiple groups was analyzed by one-way ANOVA or Kruskal–Wallis test with multiple comparisons according to the applicable conditions. The chi-squared was employed to assess the difference between grouping variables. The Pearson or Spearman test was used to examine the correlation between two variables according to the applicable conditions. All statistical tests were two-sided, and *p* value < 0.05 was judged statistically significant and labeled with *p < 0.05; **p < 0.01; ***p < 0.001.

### Reporting summary

Further information on research design is available in the Nature Research Reporting Summary linked to this article.

### Supplementary information

Supplementary files

Reporting Summary

## Data Availability

The authors are willing to provide any data upon reasonable request.

## Code availability

The bioinformatics analysis used in this work was conducted using R 4.0.2, and the R packages that were utilized were thoroughly described in the “Materials and methods” section. The authors are willing to provide any codes upon reasonable request.

## References

[1] Siegel RL, Miller KD, Fuchs HE, Jemal A (2022). Cancer statistics, 2022. CA Cancer J. Clin..

[2] Mei J (2020). Systematic characterization of non-coding RNAs in triple-negative breast cancer. Cell Prolif..

[3] Bianchini G, Balko JM, Mayer IA, Sanders ME, Gianni L (2016). Triple-negative breast cancer: challenges and opportunities of a heterogeneous disease. Nat. Rev. Clin. Oncol..

[4] Bou Zerdan M (2022). Triple negative breast cancer: updates on classification and treatment in 2021. Cancers.

[5] Howard FM, Pearson AT, Nanda R (2022). Clinical trials of immunotherapy in triple-negative breast cancer. Breast Cancer Res. Treat..

[6] Qiu D (2021). Prospects of immunotherapy for triple-negative breast cancer. Front. Oncol..

[7] Hofmeyer KA, Ray A, Zang X (2008). The contrasting role of B7-H3. Proc. Natl Acad. Sci. USA.

[8] Zhou WT, Jin WL (2021). B7-H3/CD276: an emerging cancer immunotherapy. Front. Immunol..

[9] Arigami T (2010). B7-h3 ligand expression by primary breast cancer and associated with regional nodal metastasis. Ann. Surg..

[10] Cong F, Yu H, Gao X (2017). Expression of CD24 and B7-H3 in breast cancer and the clinical significance. Oncol. Lett..

[11] Zhao B (2022). Clinical significance of the expression of co-stimulatory molecule B7-H3 in papillary thyroid carcinoma. Front. Cell Dev. Biol..

[12] Wang LC (2022). Expression and clinical significance of VISTA, B7-H3, and PD-L1 in glioma. Clin. Immunol..

[13] Lei X (2021). A pan-histone deacetylase inhibitor enhances the antitumor activity of B7-H3-specific CAR T cells in solid tumors. Clin. Cancer Res..

[14] Yonesaka K (2018). B7-H3 negatively modulates CTL-mediated cancer immunity. Clin. Cancer Res..

[15] Zhao B (2022). Immune checkpoint of B7-H3 in cancer: from immunology to clinical immunotherapy. J. Hematol. Oncol..

[16] Garcia-Mulero S (2020). Lung metastases share common immune features regardless of primary tumor origin. J. Immunother. Cancer.

[17] Braso-Maristany F (2022). Gene expression profiles of breast cancer metastasis according to organ site. Mol. Oncol..

[18] Necula L (2022). Collagen family as promising biomarkers and therapeutic targets in cancer. Int. J. Mol. Sci..

[19] Bagaev A (2021). Conserved pan-cancer microenvironment subtypes predict response to immunotherapy. Cancer Cell.

[20] Chen D (2020). Enhanced B7-H4 expression in gliomas with low PD-L1 expression identifies super-cold tumors. J. Immunother. Cancer.

[21] Cherif B (2021). Immune checkpoint molecules B7-H6 and PD-L1 co-pattern the tumor inflammatory microenvironment in human breast cancer. Sci. Rep..

[22] Deepak KGK (2020). Tumor microenvironment: challenges and opportunities in targeting metastasis of triple negative breast cancer. Pharm. Res..

[23] Mahmoud AM (2022). Evaluation of PD-L1 and B7-H3 expression as a predictor of response to adjuvant chemotherapy in bladder cancer. BMC Urol..

[24] Yang J (2022). Clinical significance and correlation of PD-L1, B7-H3, B7-H4, and TILs in pancreatic cancer. BMC Cancer.

[25] Prasad DV (2004). Murine B7-H3 is a negative regulator of T cells. J. Immunol..

[26] Wang C (2021). CD276 expression enables squamous cell carcinoma stem cells to evade immune surveillance. Cell Stem Cell.

[27] Zhang S, Zhou C, Zhang D, Huang Z, Zhang G (2019). The anti-apoptotic effect on cancer-associated fibroblasts of B7-H3 molecule enhancing the cell invasion and metastasis in renal cancer. Onco Targets Ther..

[28] Cheng N (2021). B7-H3 augments the pro-angiogenic function of tumor-associated macrophages and acts as a novel adjuvant target for triple-negative breast cancer therapy. Biochem. Pharm..

[29] Kim NI, Park MH, Cho N, Lee JS (2022). Comparison of the clinicopathologic features and T-cell infiltration of B7-H3 and B7-H4 expression in triple-negative breast cancer subtypes. Appl. Immunohistochem. Mol. Morphol..

[30] Li H, van der Merwe PA, Sivakumar S (2022). Biomarkers of response to PD-1 pathway blockade. Br. J. Cancer.

[31] Tan Q (2022). Potential predictive and prognostic value of biomarkers related to immune checkpoint inhibitor therapy of triple-negative breast cancer. Front. Oncol..

[32] Mittendorf EA (2020). Neoadjuvant atezolizumab in combination with sequential nab-paclitaxel and anthracycline-based chemotherapy versus placebo and chemotherapy in patients with early-stage triple-negative breast cancer (IMpassion031): a randomised, double-blind, phase 3 trial. Lancet.

[33] Savas P, Loi S (2020). Expanding the role for immunotherapy in triple-negative breast cancer. Cancer Cell.

[34] Lu S (2019). Comparison of biomarker modalities for predicting response to PD-1/PD-L1 checkpoint blockade: a systematic review and meta-analysis. JAMA Oncol..

[35] Hu G (2022). Case report: B7-H3 CAR-T therapy partially controls tumor growth in a basal cell carcinoma patient. Front. Oncol..

[36] Tang X (2021). Administration of B7-H3 targeted chimeric antigen receptor-T cells induce regression of glioblastoma. Signal Transduct. Target Ther..

[37] Xu K (2021). Single-cell RNA sequencing reveals cell heterogeneity and transcriptome profile of breast cancer lymph node metastasis. Oncogenesis.

[38] Butler A, Hoffman P, Smibert P, Papalexi E, Satija R (2018). Integrating single-cell transcriptomic data across different conditions, technologies, and species. Nat. Biotechnol..

[39] Korsunsky I (2019). Fast, sensitive and accurate integration of single-cell data with Harmony. Nat. Methods.

[40] Zang X (2010). Tumor associated endothelial expression of B7-H3 predicts survival in ovarian carcinomas. Mod. Pathol..

[41] MacGregor HL (2019). High expression of B7-H3 on stromal cells defines tumor and stromal compartments in epithelial ovarian cancer and is associated with limited immune activation. J. Immunother. cancer.

[42] Costa A (2018). Fibroblast heterogeneity and immunosuppressive environment in human breast cancer. Cancer Cell.

[43] Chen C (2013). Induced expression of B7-H3 on the lung cancer cells and macrophages suppresses T-cell mediating anti-tumor immune response. Exp. Cell Res..

[44] Salgado R (2015). The evaluation of tumor-infiltrating lymphocytes (TILs) in breast cancer: recommendations by an International TILs Working Group 2014. Ann. Oncol..

[45] Cancer Genome Atlas Network. (2015). Genomic classification of cutaneous melanoma. Cell.

[46] Cerami E (2012). The cBio cancer genomics portal: an open platform for exploring multidimensional cancer genomics data. Cancer Discov..

[47] Rose TL (2021). Fibroblast growth factor receptor 3 alterations and response to immune checkpoint inhibition in metastatic urothelial cancer: a real world experience. Br. J. Cancer.

[48] Wolf DM (2022). Redefining breast cancer subtypes to guide treatment prioritization and maximize response: predictive biomarkers across 10 cancer therapies. Cancer Cell.

[49] Blenman KRM (2022). Predictive markers of response to neoadjuvant durvalumab with Nab-paclitaxel and dose-dense doxorubicin/cyclophosphamide in basal-like triple-negative breast cancer. Clin. Cancer Res..

[50] de Ronde JJ (2013). SERPINA6, BEX1, AGTR1, SLC26A3, and LAPTM4B are markers of resistance to neoadjuvant chemotherapy in HER2-negative breast cancer. Breast Cancer Res. Treat..

[51] Garcia-Recio S (2023). Multiomics in primary and metastatic breast tumors from the AURORA US network finds microenvironment and epigenetic drivers of metastasis. Nat. Cancer.

[52] Garcia-Recio S (2020). FGFR4 regulates tumor subtype differentiation in luminal breast cancer and metastatic disease. J. Clin. Invest..

[53] Leek JT, Johnson WE, Parker HS, Jaffe AE, Storey JD (2012). The sva package for removing batch effects and other unwanted variation in high-throughput experiments. Bioinformatics.

[54] Johnson WE, Li C, Rabinovic A (2007). Adjusting batch effects in microarray expression data using empirical Bayes methods. Biostatistics.

[55] Georgiev P (2022). Reverse translating molecular determinants of anti-programmed death 1 immunotherapy response in mouse syngeneic tumor models. Mol. Cancer Ther..

[56] Liberzon A (2015). The molecular signatures database (MSigDB) hallmark gene set collection. Cell Syst..

[57] Xu L (2018). TIP: a web server for resolving tumor immunophenotype profiling. Cancer Res..

