# Supplementary material for: High B7-H3 expression with low PD-L1 expression identifies armored-cold tumors in triple-negative breast cancer
Source: NPJ Breast Cancer. 2024 Jan 27;10:11. doi: 10.1038/s41523-024-00618-6 (PMC10821876; doi:10.1038/s41523-024-00618-6)
Supplement: Supplementary file 1 — Supplementary files [file 41523_2024_618_MOESM1_ESM.pdf]

Supplementary files

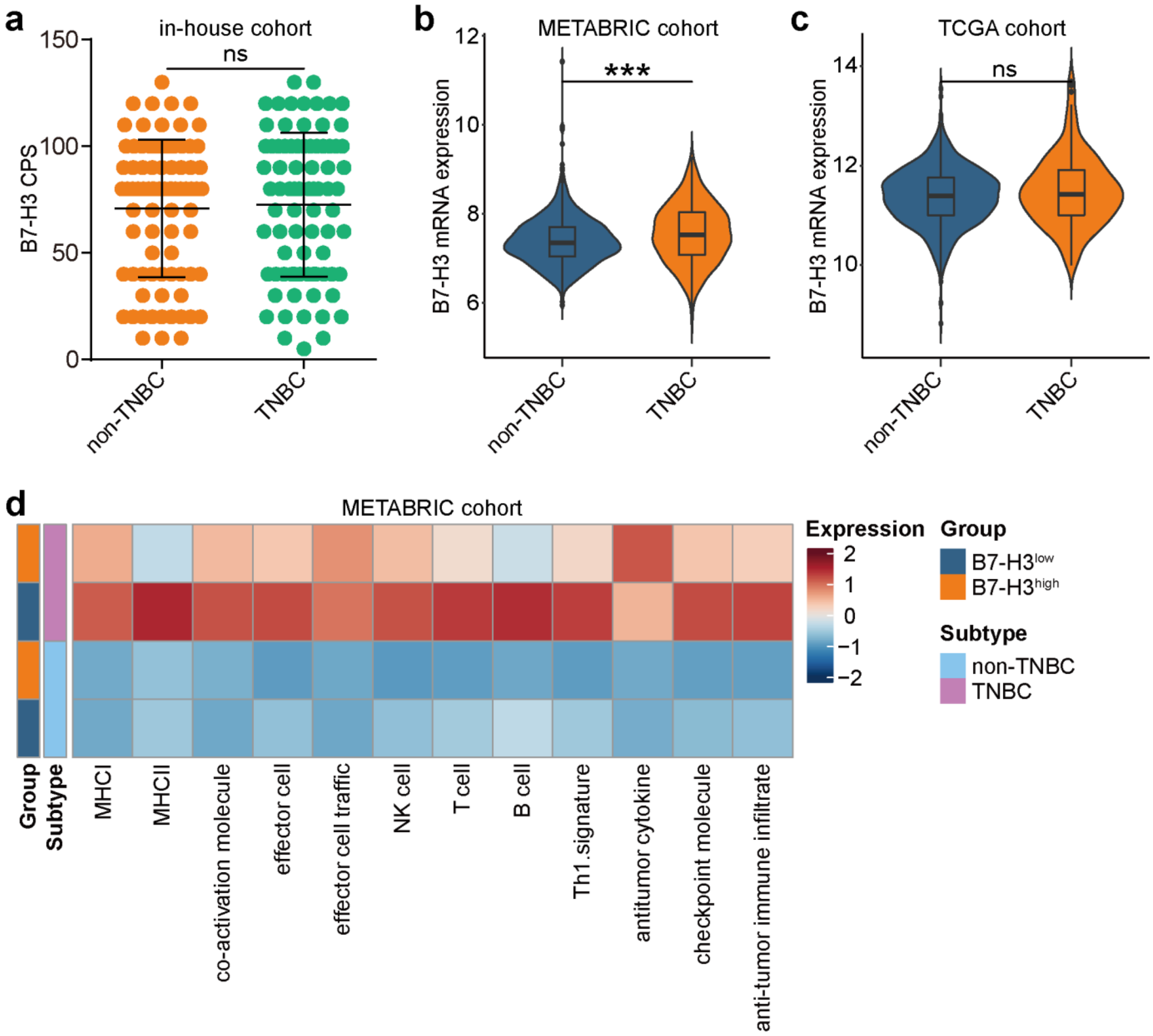

Supplementary Figure 1. Expression of B7-H3 in TNBC and non-TNBC samples. (a-c) B7-H3 expression in TNBC and non-TNBC samples. a: The in-house cohort, Data presented as mean ± SD. b: The TCGA cohort. c: The METABRIC cohort. Significance was calculated with Student's t test. (d) Anti-tumor immune infiltration levels in non-TNBC and TNBC samples with the low and high B7-H3 expression in the METABRIC cohort.

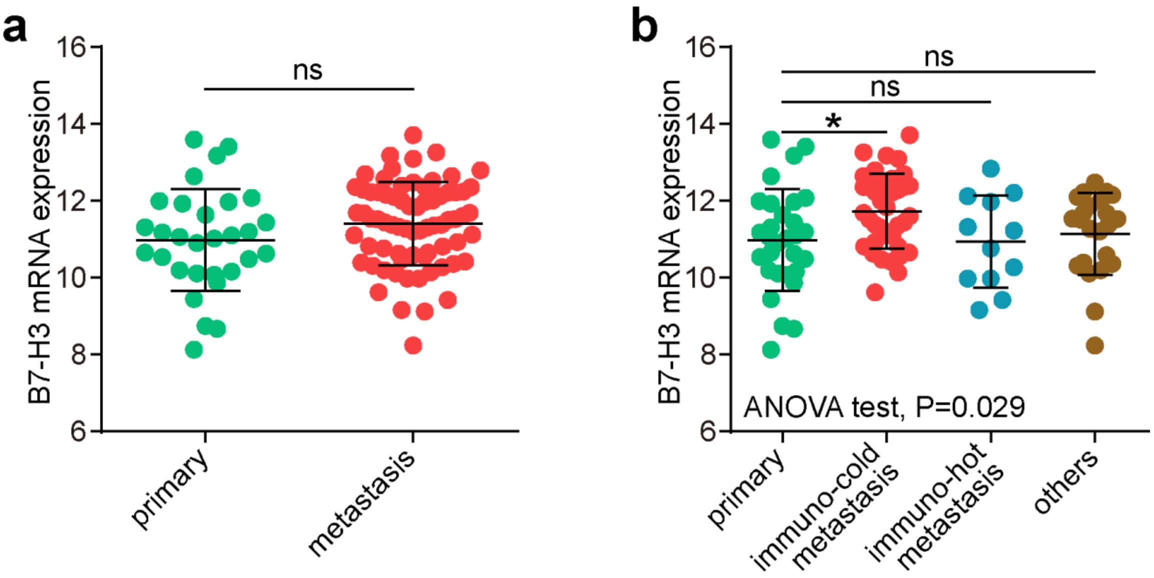

Supplementary Figure 2. Expression of B7-H3 in primary and metastatic TNBC tissues. (a) Expression of B7-H3 in primary and metastatic tissues at all organs. Data presented as mean ± SD. Significance was calculated with Student's t test. (b) Expression of B7-H3 in primary and immuno-hot metastasis, immuno-cold metastasis, and other metastasis. Data presented as mean ± SD. Significance was calculated with 1-way ANOVA with Tukey's multiple-comparison test.

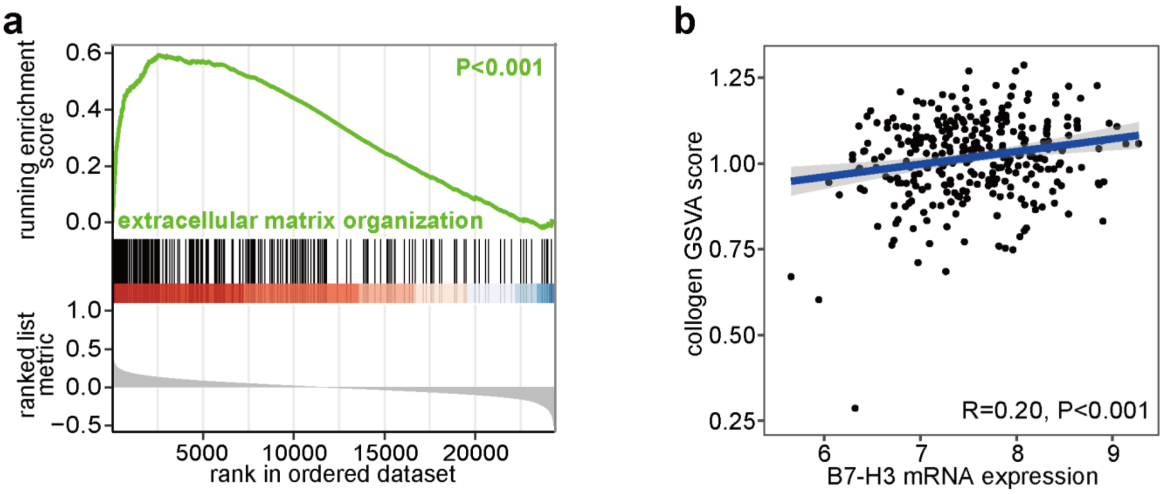

Supplementary Figure 3. Correlation between B7-H3 and collagen deposition in the METABRIC cohort. (a) GSEA revealing the association between B7-H3 and extracellular matrix organization. (b) Correlation between B7-H3 and collagen gene expression & collagen GSVA score. Significance was calculated with Pearson test.

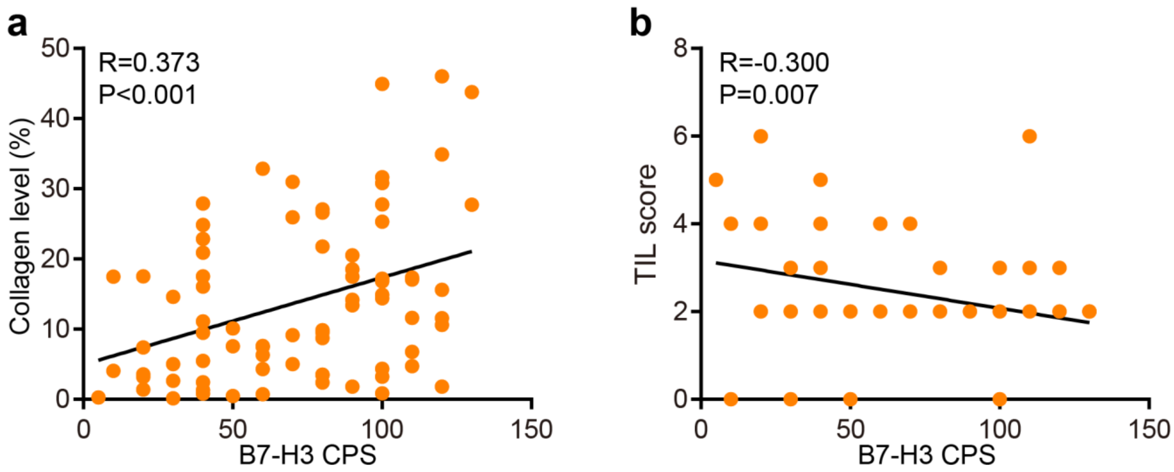

Supplementary Figure 4. Correlation between B7-H3 expression and collagen level &TIL score in TNBC. (a) Positive correlation between B7-H3 expression and collagen levels. Significance was calculated with Pearson test. (b) Negative correlation between B7-H3 expression and immune cells infiltration. Significance was calculated with Spearman test.

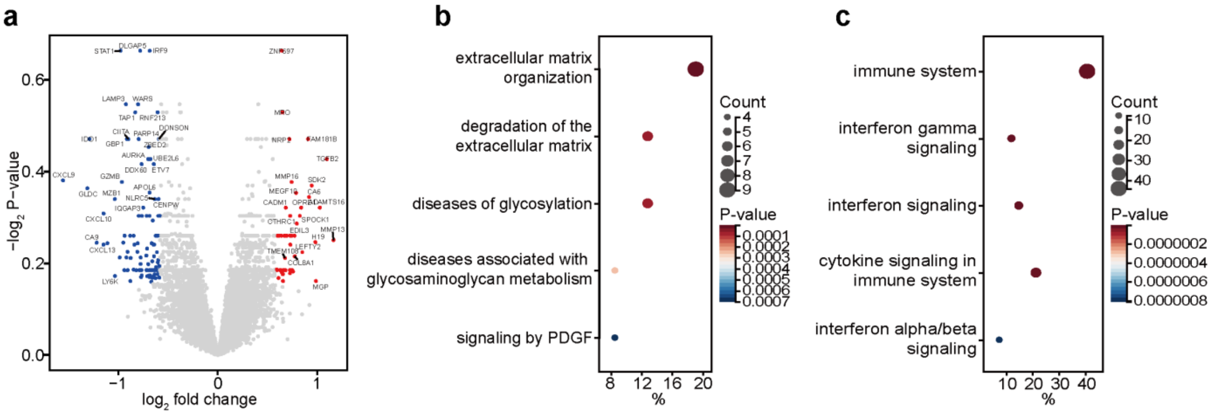

Supplementary Figure 5. Response-related genes in the merged durvalumab-based therapy cohort and related functions. (a) Identification of response-related genes. (b) Enrichment of genes upregulated in the patients with poor responses. (c) Enrichment of genes upregulated in the patients with well responses.

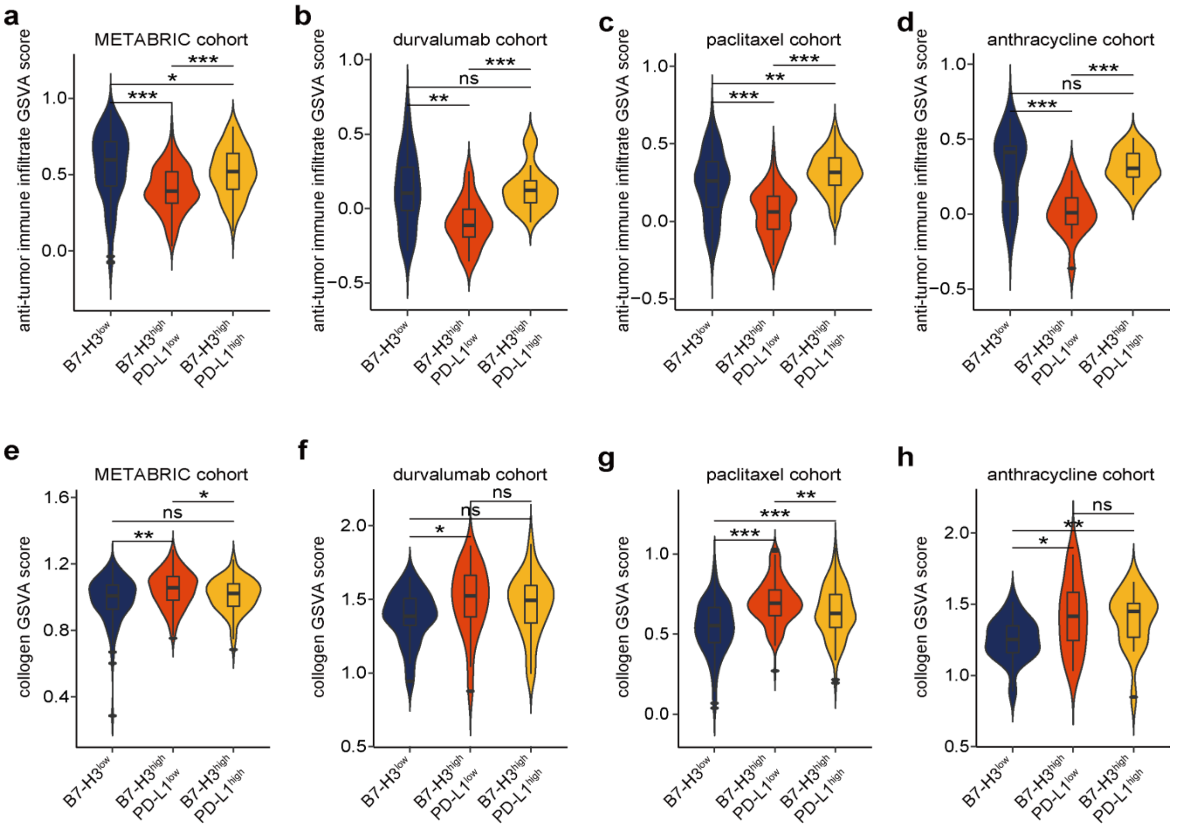

Supplementary Figure 6. TIME features of three subtypes in four different cohorts. (a-d) Violin plot showing the difference in anti-tumor immune infiltration in TNBC samples in the B7-H3^{low}, the B7-H3^{high}&PD-L1^{low}, and the B7-H3^{high}&PD-L1^{low} subtypes in the METABRIC, durvalumab, the paclitaxel, and the anthracycline cohorts. Significance was calculated with 1-way ANOVA with Tukey's multiple-comparison test. (e-h) Difference of collagen infiltration in TNBC samples in the B7-H3^{low}, the B7-H3^{high}&PD-L1^{low}, and the B7-H3^{high}&PD-L1^{high} subtypes in the METABRIC, the durvalumab, the paclitaxel, and the anthracycline cohorts. Significance was calculated with 1-way ANOVA with Tukey's multiple-comparison test.

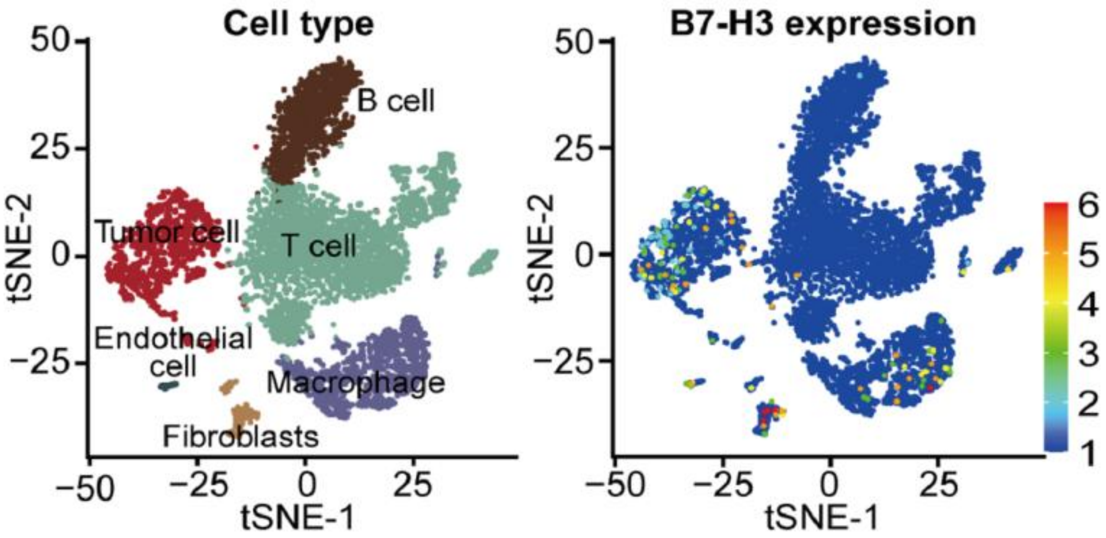

Supplementary Figure 7. t-SNE visualization of cell types in TNBC patients (the GSE180286 dataset) and B7-H3 expression.

Supplementary Table 1. Clinic-pathological parameters of patients in the recruited NAT cohort.

Clinic-pathological parameters	Case	Proportion
Age		
≤60	18	60.00%
>60	12	40.00%
T stage		
T2	20	66.67%
T3	5	16.67%
T4	5	16.67%
N stage		
N0	3	10.00%
N1	8	26.67%
N2	11	36.67%
N3	8	26.67%
M stage		
M0	28	93.33%
M1	2	6.67%
Miller-Payne grade		
1	1	3.33%
2	5	16.67%
3	8	26.67%
4	5	16.67%
5	9	30.00%
unknown	2	6.67%
Ki-67 positive rate		
≤60%	14	46.67%
>60%	16	53.33%

Supplementary Table 2. Public and in-house cohorts included in the current research.

Cohort	Case (TNBC case)	Source
Public cohort		
BC-METABRIC	1904 (298)	http://www.cbioportal.org/
BC-TCGA	1104 (145)	http://xenabrowser.net/datapages/
GSE173839	71 (21)	http://www.ncbi.nlm.nih.gov/geo/
PRJNA558949	50 TNBC	http://www.ncbi.nlm.nih.gov/bioproject/
GSE194040	988 (362)	http://www.ncbi.nlm.nih.gov/geo/
GSE34138	178 (55)	http://www.ncbi.nlm.nih.gov/geo/
GSE168846	21 mouse tumors	http://www.ncbi.nlm.nih.gov/geo/
GSE209998<br>GSE193103<br>GSE147322	30 primary<br>72 metastasis	http://www.ncbi.nlm.nih.gov/geo/
In-house cohort		
HBreD090Bc01	80 TNBC	Outdo BioTech
HBreD090Bc03	83 non-TNBC	Outdo BioTech
NAT cohort	30 TNBC	recruitment from multi-center
