# Supplementary material for: High B7-H3 expression with low PD-L1 expression identifies armored-cold tumors in triple-negative breast cancer
Source: NPJ Breast Cancer. 2024 Jan 27;10:11. doi: 10.1038/s41523-024-00618-6 (PMC10821876; doi:10.1038/s41523-024-00618-6)
Supplement: Supplementary file 2 — Reporting Summary [file 41523_2024_618_MOESM2_ESM.pdf]

nature portfolio

Corresponding author(s): Yongmei Yin, Wenjun Mao, Yan Zhang

Last updated by author(s): Aug 5, 2023

Reporting Summary

Nature Portfolio wishes to improve the reproducibility of the work that we publish. This form provides structure for consistency and transparency in reporting. For further information on Nature Portfolio policies, see our Editorial Policies and the Editorial Policy Checklist.

Statistics

For all statistical analyses, confirm that the following items are present in the figure legend, table legend, main text, or Methods section.

n/a	Confirmed	
☐	☒	The exact sample size (*n*) for each experimental group/condition, given as a discrete number and unit of measurement
☐	☒	A statement on whether measurements were taken from distinct samples or whether the same sample was measured repeatedly
☐	☒	The statistical test(s) used AND whether they are one- or two-sided<br>*Only common tests should be described solely by name; describe more complex techniques in the Methods section.*
☐	☒	A description of all covariates tested
☐	☒	A description of any assumptions or corrections, such as tests of normality and adjustment for multiple comparisons
☐	☒	A full description of the statistical parameters including central tendency (e.g. means) or other basic estimates (e.g. regression coefficient) AND variation (e.g. standard deviation) or associated estimates of uncertainty (e.g. confidence intervals)
☐	☒	For null hypothesis testing, the test statistic (e.g. *F*, *t*, *r*) with confidence intervals, effect sizes, degrees of freedom and *P* value noted<br>*Give P values as exact values whenever suitable.*
☒	☐	For Bayesian analysis, information on the choice of priors and Markov chain Monte Carlo settings
☒	☐	For hierarchical and complex designs, identification of the appropriate level for tests and full reporting of outcomes
☒	☐	Estimates of effect sizes (e.g. Cohen's *d*, Pearson's *r*), indicating how they were calculated

Our web collection on statistics for biologists contains articles on many of the points above.

Software and code

Policy information about availability of computer code

Data collection	GSE180286, TCGA, METABRIC, GSE176307, GSE194040, PRJNA558949, GSE34138, GSE147322, GSE209998, GSE193103, GSE168846
Data analysis	R, Graphpad Prism, HALO

For manuscripts utilizing custom algorithms or software that are central to the research but not yet described in published literature, software must be made available to editors and reviewers. We strongly encourage code deposition in a community repository (e.g. GitHub). See the Nature Portfolio guidelines for submitting code & software for further information.

Data

Policy information about availability of data

All manuscripts must include a data availability statement. This statement should provide the following information, where applicable:

- Accession codes, unique identifiers, or web links for publicly available datasets
- A description of any restrictions on data availability
- For clinical datasets or third party data, please ensure that the statement adheres to our policy

The authors are willing to provide any data upon reasonable request.

Research involving human participants, their data, or biological material

Policy information about studies with human participants or human data. See also policy information about sex, gender (identity/presentation), and sexual orientation and race, ethnicity and racism.

Reporting on sex and gender	For the breast cancer, all the patients enrolled are female.
Reporting on race, ethnicity, or other socially relevant groupings	There are no race discrepancy. All the patients are Chinese from four clinical center of China.
Population characteristics	Table S1, Table S2
Recruitment	The paraffin-embedded tissue microarrays (TMAs) of non-TNBC (Cat. HBreD090Bc03) and TNBC (Cat. HBreD090Bc01) were provided by Outdo BioTech (Shanghai, China). Outdo BioTech provided detailed clinic-pathological features. Samples that were exfoliated during immunohistochemistry (IHC), Hematoxylin and Eosin (HE), and masson staining were removed. A total of 163 samples (83 non-TNBC and 80 TNBC) were included in this study. The HE staining was reviewed by two pathologists, and confirmed 6 samples were confirmed to include paired para-tumor samples. In addition, 30 TNBC patients receiving standardized neoadjuvant chemotherapy (NAT) were recruited from four independent medical units. In addition, the responses were assessed using the RECIST1.1 criterion after receiving 8 cycles of NAT. The paraffin-embedded samples of these patients before receiving NAT were obtained. Table S1 lists the specific clinic-pathological characteristics.
Ethics oversight	The Clinical Research Ethics Committee of Outdo Biotech granted ethical approval for the use of TMAs. Ethical approval was granted by the involved institutions and informed consent was obtained.

Note that full information on the approval of the study protocol must also be provided in the manuscript.

Field-specific reporting

Please select the one below that is the best fit for your research. If you are not sure, read the appropriate sections before making your selection.

☒ Life sciences ☐ Behavioural & social sciences ☐ Ecological, evolutionary & environmental sciences

For a reference copy of the document with all sections, see nature.com/documents/nr-reporting-summary-flat.pdf

Life sciences study design

All studies must disclose on these points even when the disclosure is negative.

Sample size	163
Data exclusions	No data exclusions
Replication	No replications. 6 replications in animal study.
Randomization	All the patients are randomly enrolled
Blinding	No blinding. This retrospective study should collect all the data from patients

Reporting for specific materials, systems and methods

We require information from authors about some types of materials, experimental systems and methods used in many studies. Here, indicate whether each material, system or method listed is relevant to your study. If you are not sure if a list item applies to your research, read the appropriate section before selecting a response.

Materials & experimental systems

n/a	Involved in the study	
☐	☒	Antibodies
☐	☒	Eukaryotic cell lines
☒	☐	Palaeontology and archaeology
☐	☒	Animals and other organisms
☒	☐	Clinical data
☒	☐	Dual use research of concern
☒	☐	Plants

Methods

n/a	Involved in the study	
☒	☐	ChIP-seq
☒	☐	Flow cytometry
☒	☐	MRI-based neuroimaging

Antibodies

Antibodies used	Anti-B7-H3 (Cat. ab219648, Abcam, Cambridge, UK), anti-PD-L1 (Cat. GT2280, GeneTech, Shanghai, China), anti-CD8 (Cat. PA067, Abcarta, Suzhou, China), anti-CD8 antibody (Cat. ab217344, Abcam)
Validation	The validation of antibodies are detailed and documented on Abcam, GeneTech, and Abcarta's websites.

Eukaryotic cell lines

Policy information about cell lines and Sex and Gender in Research

Cell line source(s)	4T1 (Cat. KG338, KeyGENE)
Authentication	NA
Mycoplasma contamination	Cel lines were routinely tested for mycoplasma
Commonly misidentified lines (See ICLAC register)	No commonly misidentified lines were used

Animals and other research organisms

Policy information about studies involving animals; ARRIVE guidelines recommended for reporting animal research, and Sex and Gender in Research

Laboratory animals	female BALB/C mice
Wild animals	Five to six week old, animals were euthanized using an extra 0.5% sodium pentobarbital solution
Reporting on sex	female
Field-collected samples	NA
Ethics oversight	All animal experiments were approved by the Laboratory Animal Ethics Committee at Nanjing Medical University.

Note that full information on the approval of the study protocol must also be provided in the manuscript.
